# Supplementary material for: Validation and Test of Measurement Invariance of the Adapted Health Consciousness Scale (HCS-G)
Source: Int J Environ Res Public Health. 2021 Jun 4;18(11):6044. doi: 10.3390/ijerph18116044 (PMC8199981; doi:10.3390/ijerph18116044)
Supplement: Supplementary file 1 [file ijerph-18-06044-s001.zip › ijerph-1223813-supplementary.pdf]

Table S1. Original and translated items of the health consciousness scale

Scoring of each item: On a 5-point Likert scale from 1="strongly disagree" to 5="strongly agree"

Item code	Original item	Translated item
hcon1C	I reflect about my health a lot.	Ich denke viel über meine Gesundheit nach.
hcon2C	I'm very self-conscious about my health.	Ich beschäftige mich bewusst mit meiner Gesundheit.
hcon3C	I'm generally attentive to my inner feeling about my health.	In Bezug auf meine Gesundheit achte ich auf meine Körpersignale.
hcon4I	I'm constantly examining my health.	Ich beobachte regelmäßig meinen Gesundheitszustand.
hcon5A	I'm alert to changes in my health.	Ich achte auf Veränderungen meines Gesundheitszustands.
hcon6A	I'm usually aware of my health.	Normalerweise bin ich mir bewusst, wie es mir gesundheitlich geht.
hcon7M	I'm aware of the state of my health as I go through the day.	Im Verlauf des Tages mache ich mir meinen Gesundheitszustand bewusst.
hcon8M	I notice how I feel physically as I go through the day.	Im Verlauf des Tages achte ich darauf, wie es mir körperlich geht.
hcon9I	I'm very involved with my health.	Ich setze mich aktiv mit meiner Gesundheit auseinander.
